# Machine learning-driven development of a novel unfolded protein response-related gene signature for predicting lung adenocarcinoma patient prognosis

**DOI:** 10.1186/s12885-026-16477-2

**Published:** 2026-07-04

**Authors:** Rui Jiao, Chengyang Wu, Tao Zhang, Hanyu Yan, Weidong He, Zhaoyang Wang, Xiaolong Yan

**Affiliations:** 1 School of Medicine, Northwest University, 229 Taibai North Road, Xi’an, 710069 China; 2 Department of Thoracic Surgery, Tangdu Hospital, The Fourth Military Medical University, Xi’an, China

**Keywords:** Lung adenocarcinoma, Unfolded protein response, Prognostic signature, Machine learning, Risk stratification

## Abstract

Lung adenocarcinoma (LUAD) is the most common lung cancer histological subtype. Although the unfolded protein response (UPR) has been linked to various human diseases, its role in LUAD remains unclear. To identify UPR-related genes, we applied various methods, including weighted gene co-expression network analysis, differential expression analysis, and multivariate Cox regression. Ten machine learning algorithms were used to construct a UPR-related signature (UPRRS), which was validated using multiple public LUAD datasets. The UPRRS was integrated into a nomogram used in clinical practice for prognosis prediction. We also evaluated predicted drug sensitivity patterns across different risk subgroups. We identified 33 UPR-associated hub genes. A UPRRS was developed through systematic evaluation of 101 machine-learning combinations, exhibiting stable prognostic performance across multiple cohorts. Integration of the UPRRS into a nomogram facilitated the construction of a quantitative prognostic model. Significant differences in biological processes and tumor microenvironment immune cell infiltration were observed between the high- and low-risk UPRRS groups. All five UPRRS genes (ALDH2, FKBP4, KLF4, LAIR1, SIDT2) were validated at the protein level in LUAD cell lines, and FKBP4 was further confirmed by IHC in clinical tissues. Functional experiments showed that FKBP4 knockdown inhibited proliferation, migration, and invasion of A549 and H1975 cells, supporting a potential role for FKBP4 in LUAD progression. Our UPRRS provides a promising tool for prognostic stratification and may offer additional insights into tumor immune microenvironment characterization and therapeutic response prediction in LUAD.

**Supplementary Information:**

The online version contains supplementary material available at https://doi.org/10.1186/s12885-026-16477-2.

## Introduction

Lung cancer is the primary contributor to cancer-associated mortality, responsible for approximately 18% of all global cancer-related deaths [1]. Among the various histological types, lung adenocarcinoma (LUAD) is the most prevalent form, accounting for approximately 50% of all lung cancer cases [2]. Despite advances in diagnostic and treatment methods, the lung cancer patient survival rate remains poor, with a 5-year survival rate between 10 and 20% [3, 4]. Targeted therapies and immunotherapies are among the most effective treatments for patients who are diagnosed with advanced-stage lung cancer. However, only a small proportion of patients can benefit from these therapies because of the significant heterogeneity and rapid development of therapeutic resistance associated with lung cancer [5, 6]. Therefore, the identification of new biomarkers with both high specificity and sensitivity is critical for the specific diagnosis, personalized treatment, and reliable prognosis for LUAD patients.

The unfolded protein response (UPR), a crucial signaling mechanism, serves to monitor and maintain the stability and integrity of the proteins within the endoplasmic reticulum (ER). This process is active when there is an accumulation of unfolded or misfolded proteins in the ER [7, 8]. In mammalian cells, the UPR is controlled through three essential ER-resident proteins: activating transcription factor 6 (ATF6), inositol-requiring enzyme 1 (IRE1), and protein kinase R-like endoplasmic reticulum kinase (PERK) [9]. While these proteins typically remain inactive, they become activated if proteins in the ER are unfolded or not folded properly. When activated, they can then regulate downstream signaling cascades and UPR effectors, thereby reducing the load on the ER. Tumor cells, which often experience rapid growth, are exposed to both internal and external stressors that can compromise protein equilibrium [10].

The UPR is inextricably linked to the development of a variety of human diseases, including neurological diseases, cardiovascular disorders, and cancer [11]. Notably, UPR activation is crucial in fostering drug resistance among cancer cells, highlighting its significance as a primary focus in cancer therapeutic development [12]. ATF6, IRE1, and PERK can regulate the transition between survival-promoting signaling that promotes cell death and are linked to multiple features of cancer [13]. Moreover, UPR-targeted approaches in cancer immunotherapy are becoming increasingly prominent [14]. In clinical settings, the UPR has shown promise in predicting outcomes for acute myeloid leukemia patients [15]. UPR-related markers have also been established for skin melanoma and liver cancer to forecast patient survival and treatment-related outcomes [16, 17]. Thus, the UPR stands out as a promising therapeutic target for various tumor types. A better understanding of UPR signals in oncology and investigating the potential advantages of UPR-targeting therapies may contribute to improving the effectiveness of cancer treatments.

Notably, similar prognostic signatures based on UPR-related pathways have been developed for other cancer types: for instance, a novel UPR-related signature for predicting acute myeloid leukemia (AML) prognosis [18], UPR-associated prognostic signatures for head and neck squamous cell carcinoma [19], and novel UPR-related gene signatures for bladder cancer outcome prediction [20]. In hepatocellular carcinoma, UPR-related molecular subtypes have also been linked to the immunogenic landscape and risk stratification [21], while UPR targeting has demonstrated potential in BRAF-mutant colorectal cancer with poor prognosis [22]. However, these models were developed in other cancer types and may not fully capture the interplay between tumor-intrinsic stress responses and the immune microenvironment in LUAD.

Although numerous UPR-related genes have been recognized as biomarkers that can affect tumor progression, no multigene signature has been linked to the UPR for predicting LUAD patient prognosis. To address this gap, we developed a prognostic model incorporating UPR-related genes and established a nomogram for risk assessment. Moreover, we performed expression and functional validation of FKBP4 to explore its potential role in LUAD progression.

## Methods

### Data acquisition and analysis

For analyzing LUAD data, transcriptomic information and pertinent clinical data of LUAD patients were obtained via The Cancer Genome Atlas (TCGA) database. Cases with deficient or missing clinicopathological information were excluded, leading to the establishment of a refined cohort consisting of 444 TCGA-LUAD individuals. For further mRNA expression analysis, the GSE13213 and GSE72094 datasets were obtained directly from the Gene Expression Omnibus (GEO) repository. These datasets offer prognostic details for 117 and 398 patients, respectively, which ensured a sufficient sample size. Somatic mutation data in MAF format were likewise retrieved from TCGA. The UPR-related genes were retrieved from the Molecular Signatures Database (MSigDB). Batch correction was conducted via the ComBat function from the ‘sva’ R package [23]. Notably, the TCGA dataset was not merged with GEO datasets before batch correction. The preprocessing workflow was performed sequentially as follows:

1. TCGA RNA-seq FPKM expression data from LUAD patients were processed independently with log2 transformation, and ComBat correction was not applied to this cohort;
2. Each GEO microarray dataset was normalized individually, merged into one unified expression matrix, log2-transformed, and then corrected for intra-platform batch effects using ComBat;
3. The batch-corrected pooled GEO matrix was split into two separate validation cohorts, GSE13213 and GSE72094. All prognostic models were trained solely on the TCGA subset and validated on the separated ComBat-adjusted GEO cohorts.

### Processing and acquisition of data for single-cell RNA sequencing (scRNA-seq) analysis

Here, scRNA-seq results collected from 25 LUAD patients were obtained via Human Tumor Atlas Network (HTAN) [24]. Data analysis was conducted using the ‘Seurat’ package [25]. For the quality control screening process, cells with mitochondrial gene percentages greater than 20% were discarded, while low-quality and sparsely expressed genes were removed before downstream analysis. The 2,000 most variable genes were subsequently selected for downstream analysis. These quality control and preprocessing procedures were implemented according to previous LUAD scRNA-seq studies [26, 27]. The ‘Harmony’ tool [28]was used to adjust for the effects of batching across the 25 samples. Cell clusters were identified using the FindNeighbors and FindClusters functions with a resolution of 0.5, then visualized using t-distributed stochastic neighbor embedding (t-SNE). Specific gene expression levels were used to characterize the various cell types. The ‘AUCell’ tool was used to calculate the area under the curve (AUC) values of the UPR gene set in each cell.

### Hub gene identification

Univariate Cox regression analysis was employed to identify important prognostic genes in the TCGA-LUAD dataset. To investigate gene expression differences between normal and cancer samples, the ‘limma’ package [28] was used for RNA differential expression analysis with the TCGA data. Significant differentially expressed genes (DEGs) were identified using the following: adjusted *P*-value < 0.05 and Log_2_ fold change (FC) > 1. The final set of UPR-related genes was identified by combining the DEGs and those with prognostic value, as well as those identified by Weighted Gene Co-expression Network Analysis (WGCNA). This intersection strategy was applied to enhance model robustness and reproducibility, and reduce false-positive signals in subsequent modeling analysis. While it is relatively stringent to avoid false positives, this design prioritizes the stability, reliability, and reproducibility of the prognostic model, which is crucial for clinical application.

### Development and validation of a UPR-related signature (UPRRS)

To avoid unstable and biased performance estimation from a single random split, the TCGA cohort was randomly partitioned into training and testing sets 10 independent times at a 7:3 ratio. The following 10 machine learning algorithms were employed: LASSO regression, Ridge regression, and elastic net using the ‘glmnet’ package [29]; CoxBoost [30]; random survival forest (RSF) [31]; partial least squares for Cox (plsRcox) [32]; supervised principal components (SuperPC) [33]; generalized boosted regression model (GBM) [34]; and survival support vector machines (survival-SVM) [35]. We constructed all 101 possible combinations of these 10 algorithms, including both single-model and pairwise hybrid combinations of feature selection and survival models. We provided an explicit description of the composition logic for the 101 algorithm combinations: the pipeline was built upon 10 base algorithms following a systematic two-stage framework, covering all possible single-model implementations and all pairwise feature-selection-plus-modeling combinations. Exhaustive enumeration was applied to avoid confirmation bias. The 101 pipelines are composed of 10 independent single-algorithm models plus 91 unique two-stage hybrid pipelines, summing to 101 total model combinations. The Elastic Net α grid ranging from 0.1 to 0.9 was used to cover the full regularization spectrum, and three Stepwise Cox directions were adopted for comprehensive variable screening. Hyperparameters were optimized instead of directly using package default configurations. Custom hyperparameter search grids were predefined for each algorithm, and tenfold cross-validation was used to select optimal parameter values on the TCGA training cohort for fair cross-model comparison. For computationally intensive algorithms (RSF, GBM, survival-SVM), we adopted standardized parameters widely validated in survival analysis literatures to balance computational load and comparability. Complete hyperparameter settings, search ranges and tuning strategies for all base algorithms are fully documented in Supplementary Table S1-1. The complete list of all 101 single-algorithm and two-stage hybrid pipelines, with separated feature selection and survival modeling components for each pipeline, is summarized in Supplementary Table S1-2. For two-stage hybrid pipelines combining feature screening and downstream survival modeling, all feature selection and hyperparameter optimization steps were restricted to training folds only to eliminate validation data leakage. This strategy allowed systematic evaluation of feature selection-model combinations to identify the most stable prognostic pipeline. The C-index values for all 101 models across training, test, and external validation cohorts are presented in Fig. 2A for comprehensive performance comparison. Model performance was first evaluated across TCGA training, TCGA testing, and external validation cohorts (GSE13213 and GSE72094) using the concordance index (C-index). To ensure that external datasets did not influence model selection, we implemented a two-stage evaluation strategy: primary ranking was based on multi-cohort performance following Liu et al. [36], while a separate TCGA-only sensitivity analysis was independently conducted after model selection, in which all 101 models were re-ranked using only TCGA training and testing cohorts. This sensitivity analysis was used solely to assess ranking stability and was not involved in model selection (Fig. S2). The LASSO + Stepwise Cox model was selected as the final prognostic model because it ranked among the top-performing pipelines across all evaluated cohorts and showed stable ranking performance in the TCGA-only sensitivity analysis (Fig. S2).

Notably, the optimal model emerged as the combination of LASSO regression and Stepwise Cox regression, achieving an average C-index of 0.645 across cohorts and demonstrating robust predictive performance. The LASSO regression analysis was performed using the ‘glmnet’ R package [29] with tenfold cross-validation to minimize partial likelihood deviation and avoid overfitting (Fig. 3B, C). This step reduced the 33 hub genes to seven genes with non-zero coefficients. Subsequently, Stepwise Cox proportional hazards regression was applied to further refine the gene set, ultimately retaining five core genes (ALDH2, FKBP4, KLF4, LAIR1, SIDT2) for UPRRS construction (Fig. 3D). The regression coefficients of these five genes were as follows: ALDH2 (−4.089 × 10^–5^), FKBP4 (1.077 × 10^–4^), KLF4 (2.745 × 10^–4^), LAIR1 (−3.474 × 10^–4^), and SIDT2 (−3.431 × 10^–4^). The UPRRS risk score for each patient was calculated as the linear combination of the expression level of each core gene multiplied by its corresponding regression coefficient.

The TCGA training and testing datasets, along with the GSE13213 and GSE72094 cohorts, were divided into low-risk and high-risk groups. The median cutoff was derived solely from the training set and then applied to the test and external validation sets. All preprocessing steps, including normalization and scaling, were performed exclusively within the training cohort. The derived parameters were applied to testing and external validation cohorts to prevent training data leakage, while external cohorts were not involved in model parameter estimation or model fitting. Risk scores were calculated using coefficients derived solely from the training dataset. To determine whether there was a significant gap in overall survival (OS) between these groups, Kaplan–Meier survival analysis was performed using the ‘survminer’ R package [37], with the log-rank test employed to assess statistical significance (*P* < 0.05). The timeROC package [38] was used to perform receiver operating characteristic (ROC) curve analysis evaluating the UPRRS predictive power for LUAD overall survival.

Cohort-specific C-index and corresponding standard error (SE) were calculated via the survival R package under Cox regression. Time-dependent AUC for 1-, 3-, and 5-year OS was estimated with the IPCW method. Two-sided 95% confidence intervals (95% CIs) for both metrics were generated by normal approximation. We additionally computed the overall mean and inter-cohort standard deviation of C-index across all four cohorts; all relevant quantitative performance metrics are compiled in Supplementary Table 2.

### Comparison with published LUAD prognostic signatures

We systematically screened published LUAD prognostic signatures related to pyroptosis, cuproptosis, hypoxia, ferroptosis, autophagy and tumor immunity. Three eligible signatures were retained (Supplementary Table 3 footnote). Signatures with ≥ 50% gene overlap with UPRRS were excluded to reduce redundant comparisons. Original gene lists of included signatures were refitted with multivariate Cox regression on the TCGA-LUAD training cohort to remove analytical bias. C-index across four cohorts (TCGA Training, TCGA Testing, GSE13213, GSE72094) was calculated to compare prognostic discriminatory ability, following a previously described benchmark pipeline [36]. Partial gene sets failed full matching to Affymetrix arrays in two GEO datasets due to platform differences, which hindered complete cross-model performance assessment.

### Development of a predictive nomogram using clinical parameters and the UPRRS

Univariate and multivariate Cox regression analyses were carried out using the TCGA database to examine if the UPRRS could help independently predict LUAD patient survival outcomes. Numerous factors, such as gender, age, tumor size (T), lymph node condition (N), metastatic dispersal (M), stage of disease progression, and UPRRS risk factors, were combined to develop a predictive nomogram. The nomogram was developed using bootstrap resampling with 1,000 repetitions. The precision of the nomogram was assessed using calibration analysis and C-index analysis. Cases with missing clinicopathological information were excluded with a missing rate < 5%, and complete-case analysis was applied to minimize bias.

### Enrichment analysis

To understand the various relevant biological functions and pathways in the high-risk and low-risk groups, Gene Ontology (GO), Kyoto Encyclopedia of Genes and Genomes (KEGG), and Reactome enrichment analyses were performed using the R program ‘clusterProfiler’ [39]. The KEGG database systematically integrates associative information on genes, pathways, and diseases, serving as a core tool for deciphering the molecular mechanisms underlying biological processes [40, 41]. Additionally, Gene Set Enrichment Analysis (GSEA) was performed to uncover the potential regulation mechanisms that may be associated with the UPRRS. The genes were ordered using their log_2_ fold change values, which were computed via differential expression analysis with the ‘limma’ package [28]. All differential expression and enrichment analyses were adjusted by the Benjamini–Hochberg false discovery rate (FDR). An adjusted P-value less than 0.05 was regarded as statistically significant.

### Immune cell infiltration and immune checkpoint expression analyses

To examine the association between the UPRRS and immune cell milieu within the lung tumor microenvironment (TME), the ESTIMATE algorithm was used. Furthermore, the CIBERSORT algorithm (LM22 signature, 1000 permutations) was utilized to assess relative proportions of 22 immune cell types. To confirm the CIBERSORT results, an XCell algorithm was performed as well. The connection between risks and immune checkpoints was demonstrated through application of the ‘ggstatsplot’ package [42].

### Prediction of potential chemotherapeutic drugs

We incorporated RNA-seq data with information on drug sensitivity taken from the Genomics of Drug Sensitivity in Cancer (GDSC) database to determine the differences in reaction in patients who are classified as UPRRS-high and -low. Drug sensitivity scores for each patient were estimated using the ‘oncoPredict’ package [43].

### Quantitative real-time polymerase chain reaction (qRT-PCR)

Total RNA was extracted from two LUAD cell lines, A549 and H1975, and an epithelial normal bronchial model, HBE, using a cell-based RNA isolation kit (Solarbio R3901). The RNA was reverse transcribed into complementary DNA (cDNA) using the PrimeScript RT reagent kit (Takara DRR037A). Next, qRT-PCR assays were performed according to the manufacturer’s instructions (Solarbio R9303) with a LightCycler 96 real-time fluorescent Quantitative PCR System (Roche, 5,815,916,001). The data were analyzed using the comparative cycle threshold (Ct) method. The Ct values of each gene were normalized using the GAPDH Ct values. The relevant primer sequences are provided in Supplementary Materials 1.

### Immunohistochemistry (IHC)

Tissue samples from tumors and their corresponding non-cancerous counterparts were procured from a group of 10 LUAD patients. These individuals were diagnosed and treated at the Department of Thoracic Surgery, Tangdu Hospital of Fourth Military Medical University (Xi’an, China). For patient data, please refer to Supplementary Material 2. Tissue sections were processed with standard deparaffinization, antigen retrieval and endogenous peroxidase inactivation. After blocking, the sections were incubated with primary antibody against FKBP4 (PA5-19,057, Thermo Fisher Scientific) at a dilution of 1:1200. Phosphate buffered saline was used to prepare negative controls. The sections were then incubated with biotinylated secondary antibody and HRP-conjugated streptavidin, followed by DAB staining and hematoxylin counterstaining. Finally, the sections were dehydrated, mounted and examined under light microscopy.

### Cell culture and lentivirus infection

Human normal bronchial epithelial (HBE) and lung adenocarcinoma (A549, H1975) cells were cultured in high-glucose DMEM (Gibco) supplemented with 10% FBS (Gibco) and 1% penicillin–streptomycin (Solarbio) at 37 °C with 5% CO₂. Cells were passaged with 0.25% trypsin–EDTA (Solarbio) at 80–90% confluence, and logarithmic-phase cells were used for experiments.

Lentiviral sh-FKBP4 and sh-NC (Obio Technology, Shanghai) were used to infect A549 and H1975 cells (30–50% confluence) with 8 μg/mL polybrene (Sigma-Aldrich). 48 h after infection, cells were selected with 2 μg/mL puromycin (Sigma-Aldrich) for 7–10 days to establish stable knockdown lines. Knockdown efficiency was verified by Western blot.

### Western blot analysis

Total protein was extracted from HBE, A549 and H1975 cells using RIPA lysis buffer (Solarbio) supplemented with protease inhibitors. Protein quantification was conducted via BCA assay (Thermo Fisher Scientific). Equal protein loads were separated by 10% SDS-PAGE and transferred to PVDF membranes (Millipore). Membranes were blocked with 5% non-fat milk in TBST for 1 h at room temperature, then incubated overnight at 4 °C with primary antibodies: ALDH2 (1:1000, #78,856, CST), FKBP4 (1:1000, #PA5-19,057, Thermo Fisher Scientific), KLF4 (1:1000, #55,004–1-AP, Proteintech), LAIR1 (1:1000, #25,289, CST), SIDT2 (1:1000, #21,544–1-AP, Proteintech), GRP78 (1:1000, #PA5-34,941, Thermo Fisher Scientific), CHOP (1:1000, #MA5-50,465, Thermo Fisher Scientific), ATF4 (1:1000, #PA5-72,620, Thermo Fisher Scientific) and VINCULIN (1:2000, Servicebio). Membranes were further incubated with HRP-conjugated secondary antibody (1:5000, Servicebio) for 1 h at room temperature. Protein bands were detected using an ECL kit (Millipore) and captured with a Bio-Rad chemiluminescence imaging system.

### CCK-8 cellular viability assay

Stable cells were seeded in 96-well plates (3 × 10^3^ cells/well) with 100 μL complete DMEM. CCK-8 solution (Beyotime, Cat No. C0038) was added at 0, 24, 48 and 72 h, and incubated at 37 °C for 1.5 h. OD value at 450 nm was measured by a microplate reader (Bio-Rad). The experiment was repeated three times independently with six technical replicates each.

### Wound healing assay

Stable cells were seeded in 6-well plates to 90–100% confluence, and a straight scratch was made with a 200 μL pipette tip. After washing with PBS (Solarbio), cells were cultured in serum-free DMEM. Wound images were captured at 0, 24 and 48 h under an inverted microscope (Olympus, 100 ×). The wound healing rate was calculated accordingly.

### EdU incorporation assay

The BeyoClick™ EdU-594 Cell Proliferation Assay Kit (Beyotime, Cat No. C0078S) was used to detect cell proliferation according to the manufacturer's directions. Stable sh-FKBP4 and sh-NC cells were seeded in 24-well plates, incubated with 50 μM EdU solution for 2 h at 37 °C, then fixed, permeabilized, and stained with Apollo dye and DAPI. EdU-positive cells were counted under a Leica confocal microscope (200 ×) and calculated as percentages of total DAPI-stained cells.

### Transwell invasion assay

24-well Transwell chambers (8.0 μm pore, Corning Costar, Cat No. 3422) pre-coated with Matrigel (1:8, BD Biosciences) were used. Stable cells (1 × 10^5^/200 μL, serum-free DMEM) were seeded in the upper chamber, and 600 μL complete DMEM (10% FBS) was added to the lower chamber. After 48 h incubation, non-invaded cells were wiped off, and invaded cells were fixed with 4% paraformaldehyde, stained with 0.1% crystal violet (Solarbio), and photographed (Olympus, 100 ×). Three random fields were counted per group.

### Statistical analysis

Unless otherwise stated, in vitro experiments were performed with at least three independent biological replicates. Data were expressed as mean ± standard deviation (SD) and analyzed by GraphPad Prism 9.0. Differences between sh-FKBP4 and sh-NC groups were compared by two-tailed Student’s t-test. *P < 0.05, **P < 0.01, ***P < 0.001 and ****P < 0.0001 were considered statistically significant.

## Results

### UPR characteristics in scRNA-seq data and identification of key UPR-related hub genes

**Fig. 1:**
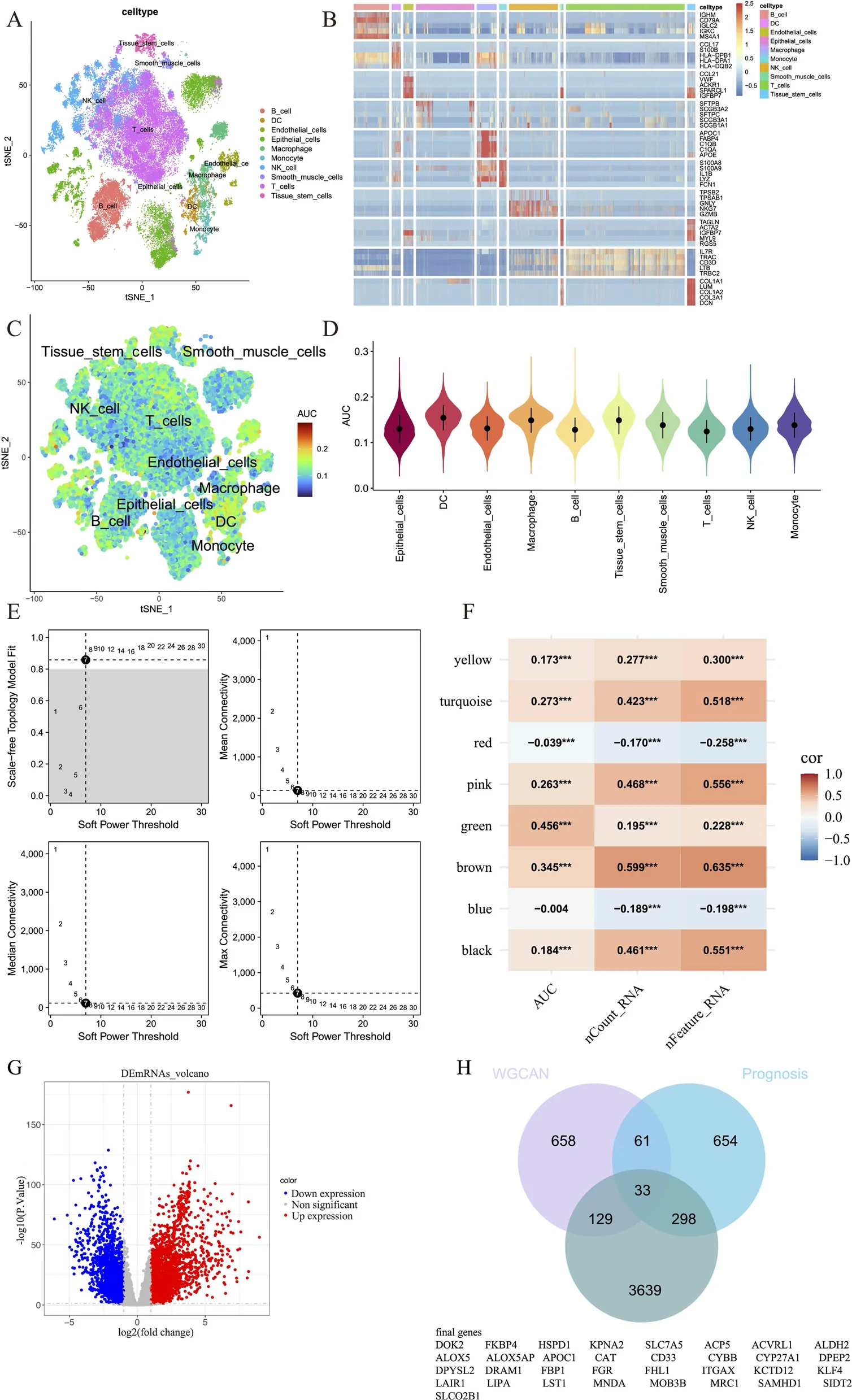
The unfolded protein response (UPR) characteristics in single‑cell transcriptomic data. **A** The t-distributed stochastic neighbor embedding (t-SNE) plot illustrates the various cell types that have been recognized using specific marker genes. **B** The heatmap depicts the top five most significant marker genes within each cell cluster. **C** The t-SNE plot illustrates the AUCell scores of the UPR gene set across different cell clusters. **D** Violin plots showing the distribution of AUCell scores across different cell clusters. **E** Determination of the optimal soft-thresholding power for hdWGCNA analysis. **F** Correlation analysis between hdWGCNA-derived gene modules and AUCell scores of UPR activity. **G** The volcano plot shows differentially expressed mRNAs between normal and tumor tissues in the TCGA-LUAD cohort. **H** The Venn diagram displays the overlap of UPR-related genes, differentially expressed genes, and prognostic genes, identifying 33 key hub genes

To characterize the UPR landscape at the single-cell level, we first analyzed the transcriptomic data of 25 LUAD patients from the HTAN database. After quality control and batch effect correction using Harmony, the 2,000 most variable genes were selected for dimensionality reduction via PCA and t-SNE. Ten major cell clusters (B_cell, dendritic cell (DC), Endothelial_cells, Epithelial_cells, Macrophage, Monocyte, natural killer (NK)_cell, Smooth_muscle_cells, T_cells, and Tissue_stem_cells) were identified using canonical marker gene expression patterns (Fig. 1A). The top five marker genes for each cell cluster were visualized using a heatmap (Fig. 1B), confirming the accuracy of cell type annotation.

To evaluate the UPR activity across different cell types, we calculated the AUC value of the UPR gene set for each cell using the ‘AUCell’ tool. The t-SNE plot (Fig. 1C) shows the spatial distribution of the AUC values, with immune cell clusters (such as Macrophage and DC) displaying intense signal. A violin plot was used to further quantify this difference. Macrophage and DC exhibited significantly higher AUC values compared with the other cell types, indicating enhanced UPR activity in these immune cell types (Fig. 1D).

Subsequently, the ‘hdWGCNA’ package was applied to identify UPR-related genes. A soft power threshold of 7 was determined to construct a stable co-expression network (Fig. 1E), which yielded eight distinct modules. Correlation analysis between the module eigengenes and AUC values revealed that the green module had the strongest association with UPR activity (Fig. 1F), leading to the selection of the 881 genes from this module as the UPR-related genes for further analysis.

The volcano plot (Fig. 1G) shows the genes with differential expression patterns between normal and malignant lung tissues within the TCGA-LUAD bulk RNA-seq data. These genes were identified by logFC > 1 and an adjusted *P*-value < 0.05. After univariate Cox regression analysis, 499 genes with prognostic significance were found in the TCGA-LUAD cohort. By intersecting the 881 genes from the green module, the DEGs from TCGA-LUAD, and the genes with prognostic significance identified by univariate Cox regression, we obtained 33 UPR-related candidate genes (Fig. 1H).

### Integrative UPRRS construction

**Fig. 2:**
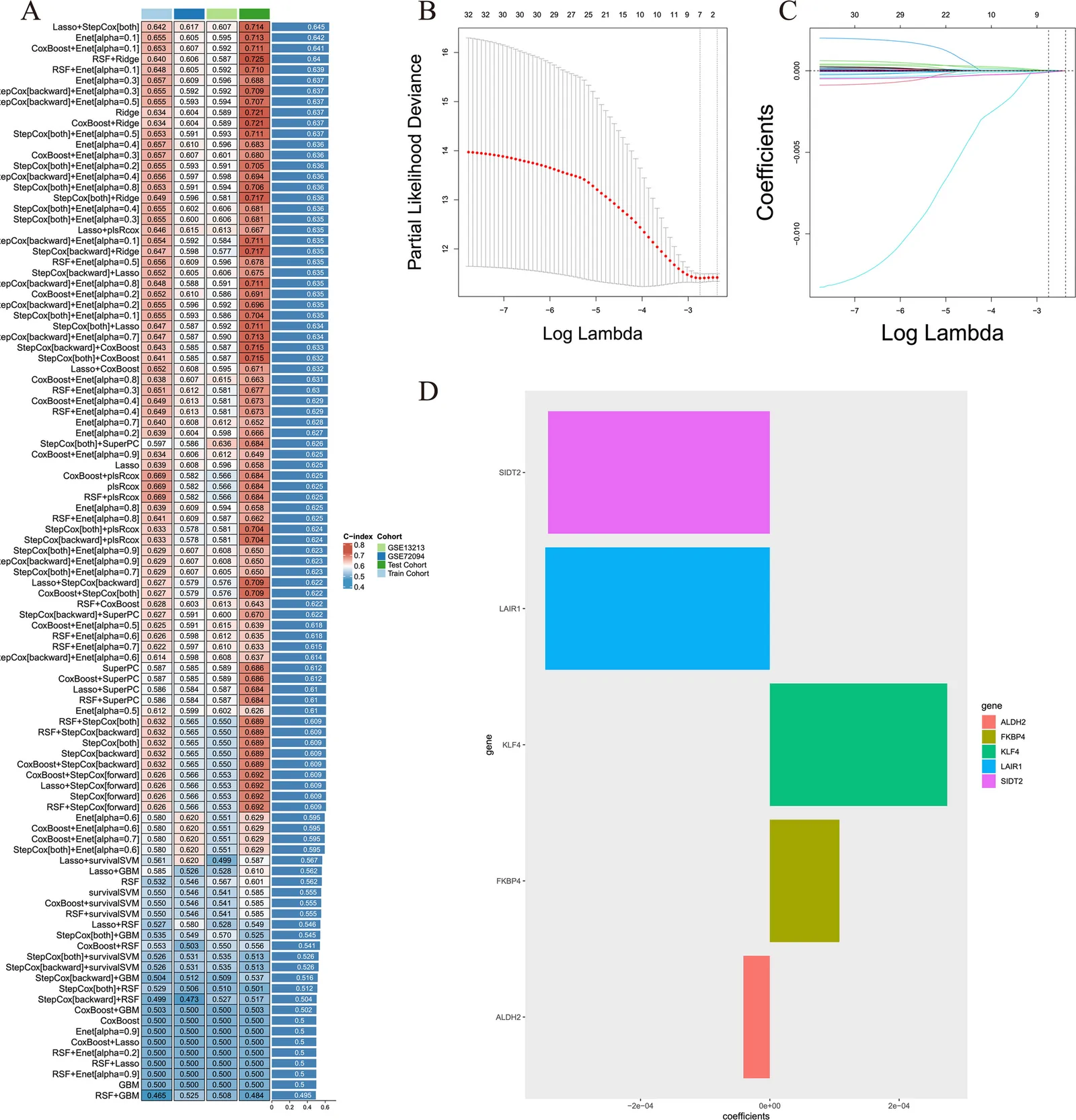
Construction of the unfolded protein response-related signature (UPRRS). **A** Heatmap showing C-index values of all 101 machine learning pipelines across training, testing, GSE13213, and GSE72094 cohorts. **B** Ten-fold cross-validation for tuning parameter selection in LASSO Cox regression. **C** LASSO coefficient profiles of candidate prognostic genes. **D** Regression coefficients of five final UPRRS genes selected by Stepwise Cox regression. Model performance was evaluated using TCGA training, TCGA testing, and external validation cohorts (GSE13213 and GSE72094) for comprehensive benchmarking purposes. To ensure that external datasets did not influence model selection, a TCGA-only sensitivity ranking analysis was independently conducted after model selection, in which all 101 models were re-ranked using only TCGA data. This sensitivity analysis was used exclusively to assess ranking stability and did not contribute to final model selection

When aiming to construct the UPRRS, we systematically evaluated 101 combinations of machine learning algorithms for model construction. These were specifically tailored to evaluate the 33 crucial genes previously identified. The TCGA-LUAD dataset was carefully divided into training and testing sets at a 7:3 ratio. For the training set, we crafted 101 predictive models, leveraging the tenfold cross-validation method. Simultaneously, we calculated the C-index for all validation and training sets, which are clearly shown in Fig. 2A. The optimal model was the combination of LASSO and Stepwise Cox regression. This model achieved a stable average C-index value of 0.645 across validation cohorts, which effectively indicated its favorable predictive ability. LASSO Cox regression identified seven prognostic genes by minimizing partial likelihood deviance (Fig. 2B, C). Genes with non-zero coefficients identified by LASSO were further refined using Stepwise Cox regression, ultimately yielding five signature genes for the final UPRRS model (Fig. 2D). Based on the original cross-cohort ranking framework, the LASSO + Stepwise Cox model was selected as the final UPRRS model. To assess the robustness of this selection strategy, a TCGA-only sensitivity ranking analysis was subsequently conducted. The LASSO + Stepwise Cox model achieved a TCGA-only mean C-index of 0.678, with merely a 0.006 gap from the best-performing pipeline across all 101 combinations (Fig. S2).

### Evaluation of the UPRRS model

**Fig. 3:**
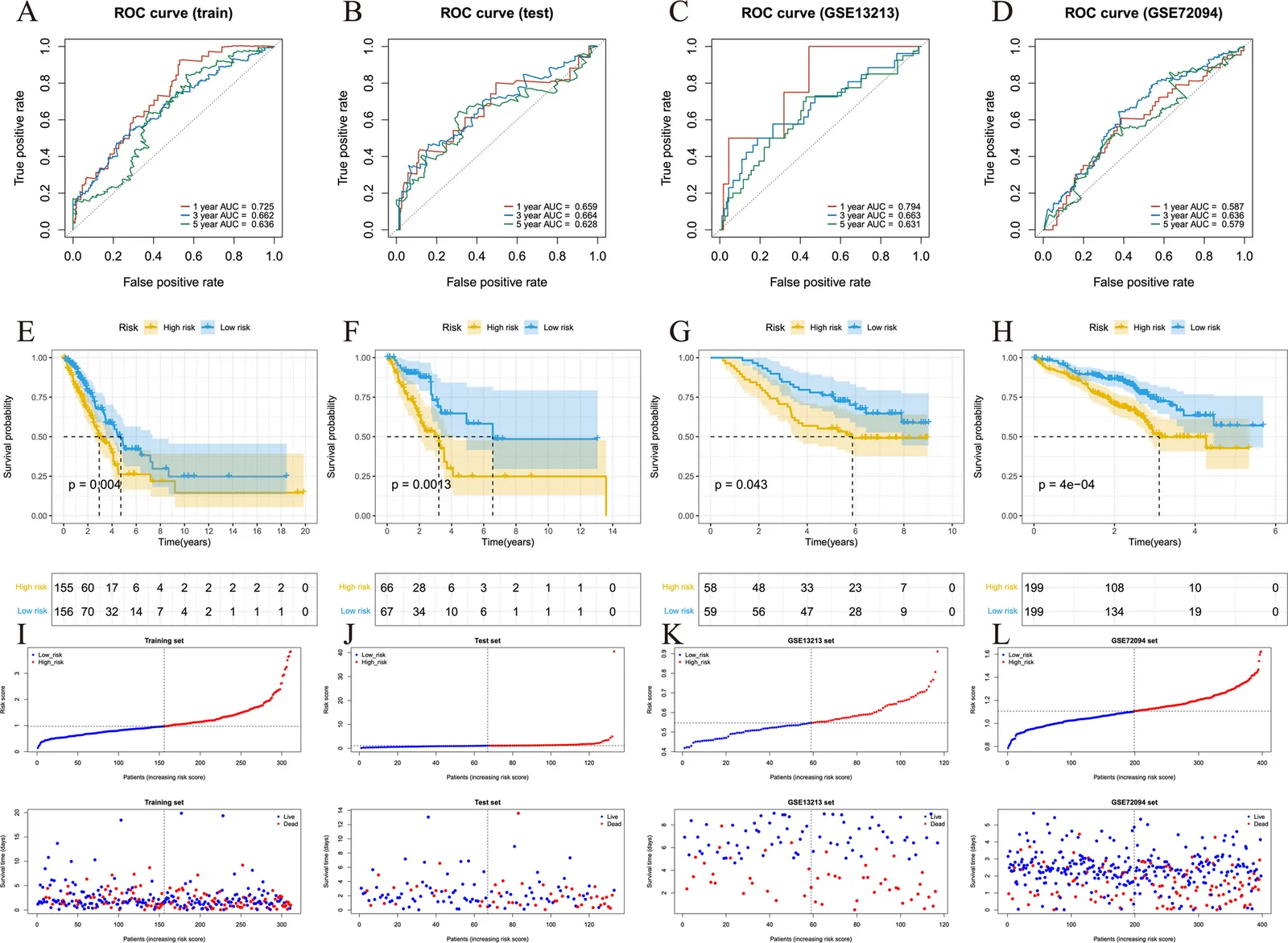
Evaluation of the unfolded protein response-related signature (UPRRS) model. **A**-**D** Time-dependent receiver operating characteristic (ROC) curves for predicting 1-, 3-, and 5-year overall survival in the training, testing, GSE13213, and GSE72094 cohorts. The curves demonstrate the prognostic value of the UPRRS. **E**–**H** Kaplan–Meier survival curves of the training cohort, testing cohort, GSE13213 cohort, and GSE72094 cohort, demonstrating survival differences between the high- and low-risk UPRRS groups. **I**-**L** Distribution of risk scores and survival status in different cohorts.

The prognostic performance of the UPRRS was evaluated using ROC analysis. The 1-year, 3-year, and 5-year AUC values revealed its predictive potential across different cohorts. As shown in Fig. 3A-D, these respective values were 0.725, 0.662, and 0.636 for the training cohort; 0.659, 0.664, and 0.628 for the testing cohort; 0.794, 0.663, and 0.631 for GSE13213; and 0.587, 0.636, and 0.579 for GSE72094. The risk score for each patient was then estimated using the expression levels of the five most important genes. These levels were weighted by the coefficients of regression within the UPRRS model. Using the median cutoff value derived from the training cohort, patients were classified into low-risk and high-risk groups. Across the training, testing, GSE13213, and GSE72094 cohorts, the high-risk patients had significantly shorter OS compared with those categorized as low-risk (log-rank test, *P* < 0.05, Fig. 3E-H). Furthermore, higher risk scores were associated with increased mortality across all cohorts (Fig. 3I-L). We further quantified model performance using cohort-specific C-index values with corresponding standard errors (SE) and 95% confidence intervals (CI). Time-dependent AUC values for 1-, 3-, and 5-year survival were also calculated with 95% CI using the IPCW method. In addition, the overall mean C-index and inter-cohort standard deviation were calculated to summarize cross-cohort stability. All detailed performance metrics, including cohort-specific C-index, SE, and 95% CI, are summarized in Supplementary Table 2.

### Multi-signature benchmark

To objectively quantify the predictive performance of UPRRS against previously reported LUAD prognostic signatures, we performed standardized head-to-head comparisons across all four cohorts using identical analytical workflows (Supplementary Table 3). Three published single-pathway signatures were enrolled for parallel evaluation, corresponding to a pyroptosis-related signature [44], an autophagy-related signature [45], and an immune-related signature [46]. All competing signatures were refitted on the same TCGA training cohort to eliminate methodological bias. In this benchmark analysis, UPRRS yielded the highest mean C-index (0.622) among all compared signatures. C-index values calculated for individual cohorts of each model are summarized in Supplementary Table 3.

### Development and evaluation of the nomogram survival model

**Fig. 4:**
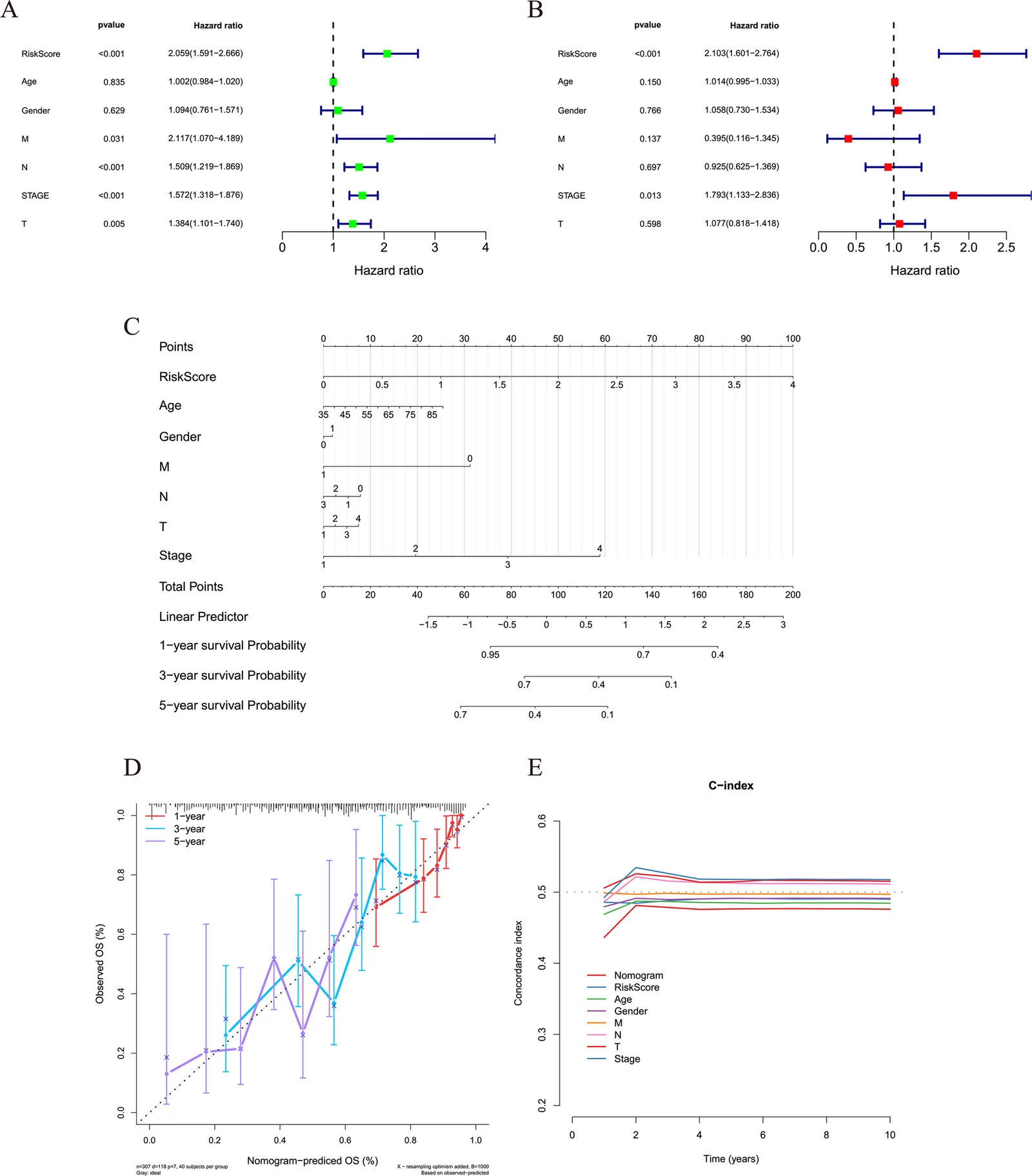
Development and evaluation of the prognostic nomogram. **A**, **B** Univariate (**A**) and multivariate (**B**) Cox regression analyses of the unfolded protein response-related signature (UPRRS) and clinicopathological variables. **C** Nomogram integrating the UPRRS and clinicopathological variables for predicting overall survival in LUAD patients. **D** Calibration curves evaluating the agreement between the predicted and observed survival probabilities of the nomogram. **E** Concordance index (C-index) analysis comparing the predictive performance of the nomogram with other clinicopathological variables

To assess the accuracy of using the UPRRS for LUAD patient survival prediction, univariate and multivariate Cox regression analyses were performed for OS using data from the TCGA-LUAD dataset (Fig. 4A, B). The results revealed that the UPRRS emerged as a highly significant risk factor for OS in both cases (hazard ratio > 1, *P* < 0.001). To increase clinical utility, we then established a nomogram, which integrated the UPRRS with other clinical variables (Fig. 4C). The calibration plots demonstrated good agreement between the predicted and observed survival probabilities (Fig. 4D). In addition, the C-index indicated favorable predictive performance of the nomogram. It demonstrated superior predictive performance compared with other clinical factors for 1- to 10-year OS prediction (Fig. 4E). These results suggest that this nomogram constructed with the UPRRS functions as a reliable model for prognostic assessment in LUAD patients.

### Enrichment analysis of the UPRRS

**Fig. 5:**
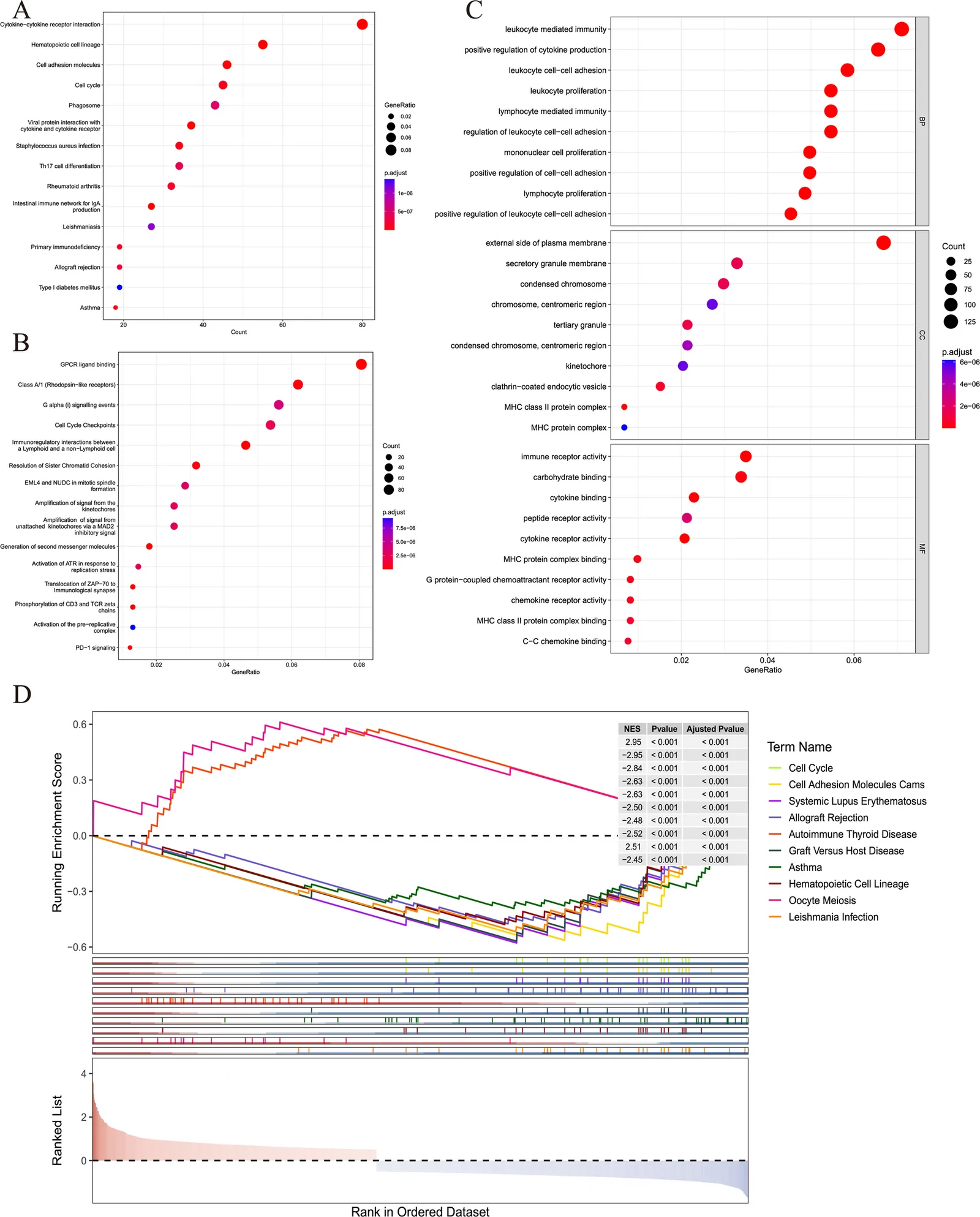
Functional enrichment analysis of the unfolded protein response-related signature (UPRRS). **A** Kyoto Encyclopedia of Genes and Genomes (KEGG) enrichment analysis of differentially expressed genes (DEGs) between the high-risk and low-risk UPRRS groups. **B** Reactome enrichment analysis. **C** GO enrichment analysis (biological process, cellular component, and molecular function). **D** Gene set enrichment analysis (GSEA)

Next, enrichment analyses were conducted to determine the functional and molecular factors that relate to both the low and high UPRRS groupings. As demonstrated in Fig. 5A, the KEGG enrichment analysis showed that the DEGs among both UPRRS groups were predominantly involved in cytokine-cytokine interactions, cell adhesion molecules, and cell cycle control. Reactome enrichment analysis identified G-protein-coupled receptor (GPCR) ligand binding, cell cycle checkpoints, and the programmed cell death protein 1 (PD-1) signaling pathways as potentially relevant to the identified differences (Fig. 5B). Additionally, GO analysis emphasized the enrichment of leukocyte-mediated immunity, the extracellular side of the plasma membrane, and immune receptor activity (Fig. 5C). GSEA indicated that the high-risk subgroup was enriched in the pathways associated with thyroid autoimmune disease and hematopoietic cell lineage (Fig. 5D). However, the low-risk subgroup had higher activity levels of pathways related to the cell cycle, adhesion proteins, and systemic lupus erythematosus. These findings provide insights into the biological processes associated with different risk groups.

### UPRRS immune characteristics

**Fig. 6:**
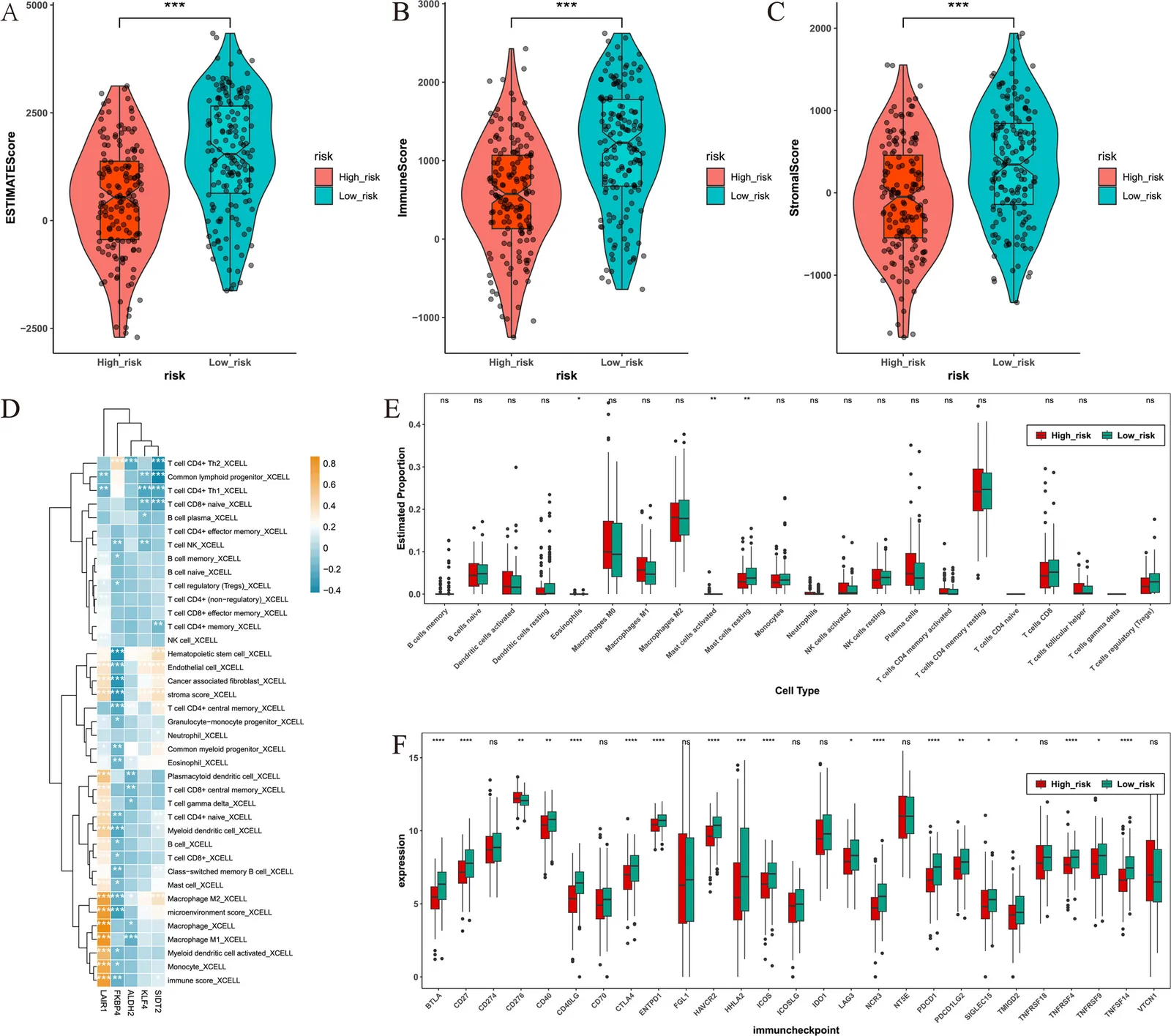
Immune landscape associated with the unfolded protein response-related signature (UPRRS). **A**-**C** Plots of the ESTIMATE, Immune, and stromal scores for quantifying tumor microenvironment complexity. **D** XCELL analysis showing the correlations between UPRRS genes and immune cell infiltration. **E** CIBERSORT analysis showing the proportions of infiltrating immune cell types in the high- and low-risk UPRRS groups. **F** Comparison of the immune checkpoint expression levels between the high-risk and low-risk UPRRS groups

To characterize immune cell infiltration patterns in LUAD samples, the ESTIMATE algorithm was applied. This enabled precise quantification of immune scores, stromal scores, ESTIMATE scores, and tumor purity across the UPRRS risk stratification groups. Notably, the low-risk UPRRS group exhibited significantly higher ESTIMATE, immune, and stromal scores, as illustrated in Fig. 6A-C. Subsequently, the XCELL algorithm was used to comprehensively investigate the correlations between UPRRS-associated genes and diverse immune cell subsets. Our findings revealed that LAIR1 expression patterns were strongly correlated with the abundance of multiple immune cell populations, whereas FKBP4 displayed negative associations with M2 macrophages, cancer-associated fibroblasts, and several other cell types (Fig. 6D). To further delineate the differences in immune cell infiltration between the high- and low-risk UPRRS groups, the CIBERSORT algorithm was applied to estimate the specific immune cell proportions in each sample (Fig. 6E). In-depth analysis demonstrated a marked enrichment of resting mast cells in the low-risk group. Moreover, except for CD276, the expression levels of immune checkpoint molecules were significantly higher in the low-risk UPRRS group than in the high-risk group (Fig. 6F). Together, these results describe the immune landscape differences associated with the UPRRS in LUAD. However, given the lack of validation cohorts treated with immunotherapy, these findings are hypothesis-generating for tumor immune landscape differences rather than for predicting clinical immunotherapy responses.

### Drug response targeting the UPRRS

To comprehensively investigate the association between our established model and drug sensitivity, we analyzed the half-maximal inhibitory concentration (IC50) values of a broad panel of compounds in LUAD samples using the GDSC database. As shown in Fig. S1 A-H, IC50 values of various drugs correlated with UPRRS status. Notably, Camptothecin, Irinotecan, Gemcitabine, Taselisib, and Mitoxantrone exhibited elevated IC50 values in the high-risk UPRRS group, suggesting differential drug response patterns between risk groups. This finding may provide insights for future personalized treatment strategies in LUAD.

However, these in silico results suggest a potential association between UPRRS-related genes and drug sensitivity. Experimental validation, such as in vitro drug viability assays, is required to confirm these observations. Therefore, conclusions regarding therapeutic sensitivity or resistance remain preliminary and should be interpreted with caution.

### Expression profiles of the five UPRRS genes and functional validation of FKBP4 in LUAD

**Fig. 7:**
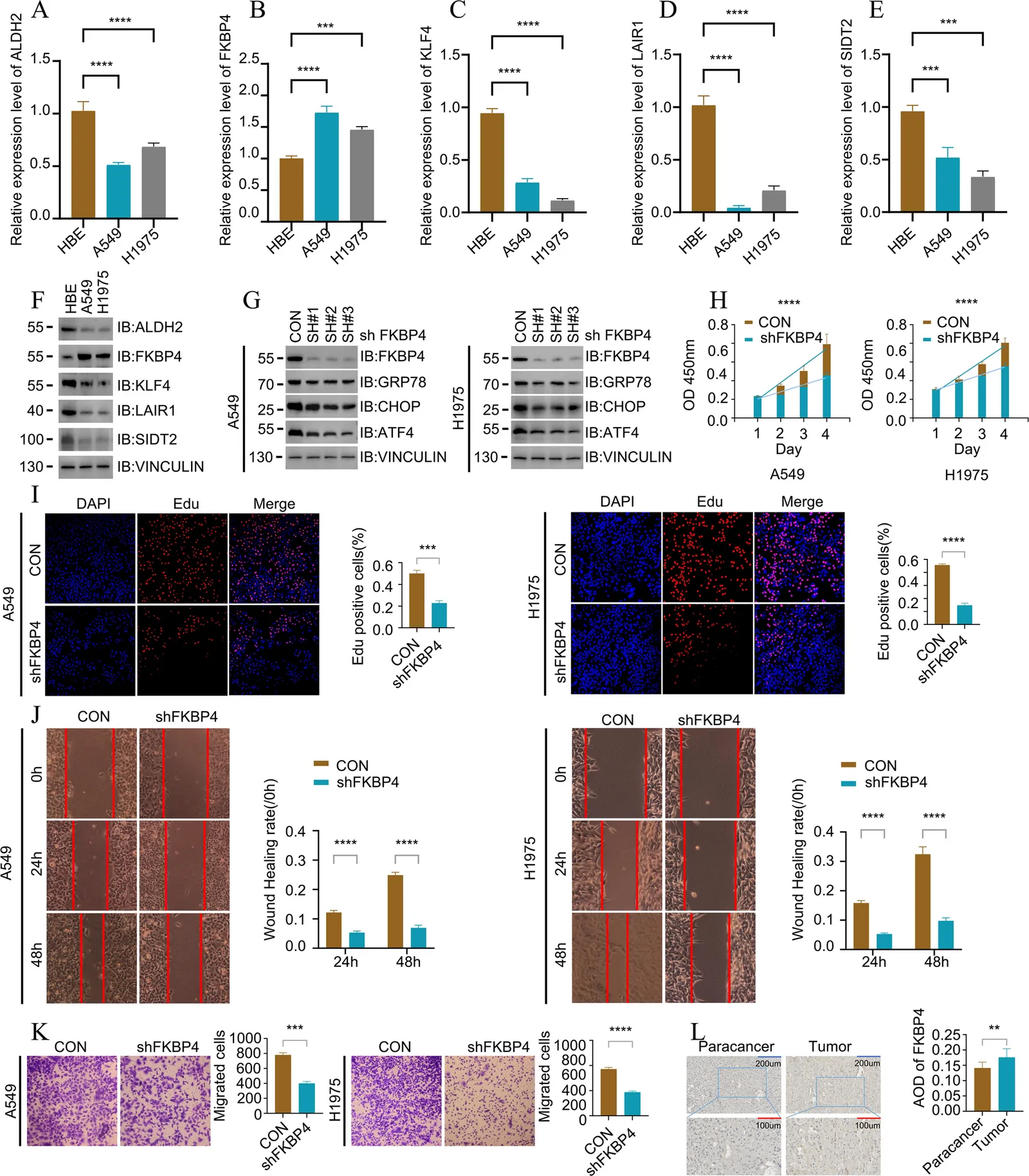
Expression characterization of five UPRRS signature genes in LUAD cell lines and functional investigation of core gene FKBP4. **A**-**E** Box plots demonstrating the mRNA expression levels of the five UPRRS genes in HBE, A549, and H1975 cells. **F** Western blot analysis of ALDH2, FKBP4, KLF4, LAIR1, and SIDT2 protein levels in HBE, A549, and H1975 cells. **G** Western blot analysis confirming stable FKBP4 knockdown efficiency in A549 and H1975 cells, as well as expression levels of representative UPR-associated proteins GRP78, ATF4, and CHOP. **H** CCK-8 assay showed that FKBP4 knockdown inhibited the proliferation of A549 and H1975 cells. **I** EdU assay verified the suppressive effect of FKBP4 silencing on cell proliferation. **J** Wound healing assay demonstrated that FKBP4 knockdown reduced cell migration capacity. **K** Transwell invasion assay indicated that silencing FKBP4 suppressed cell invasion. **L** Representative immunohistochemistry (IHC) images and quantitative analysis of FKBP4 protein expression in LUAD tissues and adjacent normal tissues (*n* = 10 paired samples). Data are presented as mean ± SD from three independent biological replicates. (*P < 0.05, **P < 0.01, ***P < 0.001, ****P < 0.0001.)

We examined the mRNA expression levels of the five UPR-related signature genes in normal HBE cells and LUAD cell lines A549 and H1975 (Fig. 7A-E). The results showed that FKBP4 was significantly upregulated at the mRNA level in LUAD cells, whereas KLF4, ALDH2, LAIR1, and SIDT2 were downregulated compared with normal control cells.

Western blot analysis generally confirmed the protein expression trends of the five genes observed at the mRNA level (Fig. 7F). FKBP4 protein was markedly increased in LUAD cells, while KLF4 protein was decreased, consistent with its well‑documented role as a tumor suppressor in lung adenocarcinoma. The other three genes (ALDH2, LAIR1, SIDT2) also showed low protein expression, in line with their status as protective genes in our UPRRS model.

Interestingly, despite the reduced expression of KLF4 in LUAD cells, multivariate Cox regression identified higher KLF4 expression as being associated with poorer prognosis in patient cohorts. This apparent inconsistency may be attributed to the complex, context‑dependent roles of KLF4 in the tumor microenvironment and UPR signaling network, rather than its cell‑autonomous function as a tumor suppressor in vitro.

Given that FKBP4 was the only gene consistently and prominently upregulated at both mRNA and protein levels in LUAD cells and served as a key high-risk factor in our five‑gene UPRRS, we selected FKBP4 for further functional validation to explore its biological role in LUAD progression.

Stable knockdown of FKBP4 was established in A549 and H1975 cells and verified by Western blot (Fig. 7G). Functional assays revealed that FKBP4 knockdown decreased overall cellular metabolic viability as measured by CCK-8 assay, and EdU incorporation assay further verified the direct inhibitory effect on cell proliferation (Fig. 7H, I). Moreover, silencing FKBP4 markedly inhibited cell migration and invasion in wound healing and Transwell invasion assays, respectively (Fig. 7J, K).

In clinical LUAD tissues, IHC further confirmed that FKBP4 protein expression was significantly higher in tumor tissues than in adjacent non‑tumor tissues (Fig. 7L).

Collectively, these data suggest that FKBP4 may contribute to oncogenic phenotypes in LUAD cells. Considering FKBP4 is one hub gene of our UPR-related prognostic signature, we further used western blot to explore the potential correlation between FKBP4 and UPR pathway activity. Figure 7G displays the protein levels of GRP78, ATF4 and CHOP in FKBP4-knockdown A549 and H1975 cells. Depletion of FKBP4 led to synchronous downregulation of these three canonical UPR markers in both cell lines, which suggests an association between FKBP4 expression and basal UPR signaling activity at the cellular level. This observation provides preliminary cellular evidence supporting the bioinformatic association between FKBP4 and the UPR pathway derived from UPRRS analysis. It should be noted that these coordinated expression changes only reflect correlation rather than definitive causal regulation, and detailed mechanistic exploration will be addressed in follow-up dedicated research.

## Discussion

The 5-year survival rate for LUAD has improved significantly in recent years, driven by the advent of novel chemotherapeutic agents, targeted therapies, and immunotherapies [47]. Despite this progress, LUAD remains a leading cause of cancer-associated morbidity and mortality worldwide. Immune checkpoint inhibitors have emerged as a promising therapeutic strategy, demonstrating notable advantages in both efficacy and safety and thus bringing renewed hope to the clinical management of non-small cell lung cancer (NSCLC) [48]. In this context, prognostic models integrating molecular features, immune microenvironment characteristics, and therapeutic response-related information may improve risk stratification and individualized treatment in LUAD [49]. Increasing evidence suggests that the UPR is involved in tumor progression, immune regulation, and therapeutic resistance across multiple cancer types [50]. However, there remains a marked paucity of validated biomarkers linked to UPR function in LUAD. In this context, we investigated the prognostic significance and potential clinical implications of a UPR-related risk signature in LUAD.

To further evaluate the predictive performance of our UPR-related prognostic signature, we compared our model with several recently published machine learning-based prognostic signatures for lung adenocarcinoma, including immune-related, intratumor heterogeneity-associated, and immune evasion-related models [26, 51, 52]. Our UPRRS achieved an average C-index of 0.645 and maintained comparable time-dependent AUC values across multiple independent cohorts, suggesting relatively stable prognostic performance.

We additionally conducted standardized head-to-head benchmarking to quantitatively compare the predictive performance of UPRRS under unified analytical criteria against three single-pathway prognostic signatures corresponding to pyroptosis [44], autophagy [45], and tumor immunity [46] (Supplementary Table 3). All competing signatures were refitted on our identical TCGA training dataset to ensure fair comparison, and UPRRS exhibited more robust prognostic discriminatory ability across all four enrolled cohorts. Unlike several previously reported signatures derived primarily from transcriptomic screening, our signature was developed based on genes associated with the UPR stress response. We further performed in vitro functional experiments to explore the association between FKBP4 and UPR signaling. We also recognize inherent limitations of this cross-signature comparison: several genes from published signatures could not be fully matched to probes on the Affymetrix microarrays of the two GEO cohorts, which restricts complete head-to-head performance evaluation across all candidate models.

Consistent with several previously published LUAD prognostic models [26, 51, 52], the predictive performance of our signature gradually declined with prolonged follow-up, with relatively better performance observed for short-term survival prediction. This phenomenon is common in transcriptome-based prognostic models and may reflect the increasing influence of complex clinical factors and dynamic tumor microenvironment changes on long-term survival outcomes. Therefore, our model may be more applicable for early prognostic evaluation and risk stratification in LUAD patients.

In contrast, our UPRRS integrates tumor-intrinsic UPR stress adaptation and immune microenvironment remodeling through five core genes identified from single-cell and bulk transcriptomic analyses. Compared with many previously reported LUAD prognostic signatures primarily based on bulk transcriptomic analyses, our study additionally incorporated single-cell transcriptomics, hdWGCNA-based UPR activity analysis, machine learning framework screening, and experimental validation. These complementary analyses may provide additional perspectives for understanding LUAD biological heterogeneity and UPR-related features.

To further explore the biological relevance of our signature, we validated core signature genes at mRNA and protein levels in LUAD cell lines and clinical specimens. Functional experiments targeting hub gene FKBP4 revealed its critical roles in cell proliferation and migration, and FKBP4 knockdown triggered coordinated expression changes of key UPR proteins, offering preliminary cellular evidence for the bioinformatic link between FKBP4 and UPR signaling. It remains unclear whether FKBP4 directly modulates the PERK-ATF4-CHOP cascade or indirectly affects UPR activity through broader cellular stress responses. Collectively, these findings support the biological relevance of UPRRS, whereas precise causal mechanisms connecting FKBP4 to UPR signaling and tumor malignant phenotypes remain unelucidated and await deeper mechanistic exploration.

Previous work has demonstrated that the UPR exerts a profound influence on the prostate cancer immune microenvironment, highlighting its potential as an innovative and promising targeted therapeutic strategy [53]. Building on this evidence, we conducted an in-depth investigation into the intricate relationship between the UPRRS and immune microenvironment in LUAD, along with its regulatory effects on tumor immunity. Our findings revealed that patients in the high-risk UPRRS group exhibited significantly lower immune scores, a phenotype that may account for their poorer clinical outcomes. In contrast, the low-risk group exhibited elevated immune checkpoint expression, pointing to distinct immunotherapy-relevant biological features across the two risk subgroups. These results reveal correlations between UPRRS and tumor immune landscape, but any inference regarding immunotherapy response remains exploratory without independent immunotherapy-treated patient cohorts for validation.

LUAD is characterized by extensive molecular heterogeneity, distinct progression patterns, and diverse biological behaviors, which pose considerable challenges for clinicians in the clinical management of affected patients [54]. Hence, optimizing personalized therapeutic approaches for LUAD is of utmost importance. Our research further explored the association between the UPRRS and predicted therapeutic sensitivity patterns in LUAD. Intriguingly, patients with higher UPRRS scores exhibited higher predicted IC50 values for several conventional chemotherapeutic agents, while potentially remaining sensitive to certain FDA-approved targeted compounds for NSCLC treatment. Compared with conventional prognostic signatures, our UPRRS specifically focuses on UPR-related pathways associated with tumor stress adaptation and therapeutic resistance, which may provide additional insights into treatment response prediction. This feature complements the predictive scope of signatures centered on metabolism or immune function. We separated preprocessing of TCGA RNA-seq and GEO microarray datasets to avoid cross-platform training-validation leakage from joint ComBat correction; cross-platform validation without unified batch correction provides a conservative test of the signature’s generalizability. In addition, a TCGA-only sensitivity ranking analysis demonstrated that the final selected model maintained comparable performance after exclusion of external validation cohorts from the ranking procedure, supporting the robustness of the model-selection strategy.

Taken together, these findings suggest that the UPRRS is associated with therapeutic response heterogeneity in LUAD and may aid prognostic evaluation and risk stratification. However, additional mechanistic research and independent clinical validation are needed to support its clinical translation.

## Supplementary Information

Supplementary Material 1.

Supplementary Material 2.

Supplementary Material 3.

Supplementary Material 4.

## Data Availability

Publicly available datasets analyzed in this study include TCGA-LUAD, GSE13213 and GSE72094 from TCGA, GEO, and HTAN databases. Other data generated during this study are included in this article and its supplementary materials. All custom R scripts, full analysis pipelines and complete parameter records are available from the corresponding author upon reasonable academic request. At present, we are organizing and standardizing all code files and auxiliary documents, and we plan to upload the sorted code repository to a public platform such as GitHub in the follow-up work.

## Abbreviations

LUAD: Lung Adenocarcinoma
UPR: Unfolded Protein Response
UPRRS: UPR-related signature
ER: Endoplasmic Reticulum
ATF6: Activating Transcription Factor 6
IRE1: Inositol-Requiring Enzyme 1
PERK: Protein kinase R-like endoplasmic reticulum kinase
TCGA: The Cancer Genome Atlas
GEO: Gene Expression Omnibus
scRNA-seq: Single-cell RNA sequencing
WGCNA: Weighted Gene Co-expression Network Analysis
DEGs: Differentially Expressed Genes
AUC: Area Under the Curve
C-index: Concordance Index
OS: Overall Survival
ROC: Receiver Operating Characteristic
GO: Gene Ontology
KEGG: Kyoto Encyclopedia of Genes and Genomes
GSEA: Gene Set Enrichment Analysis
TME: Tumor Microenvironment
qRT-PCR: Quantitative real-time polymerase chain reaction
IHC: Immunohistochemistry
GDSC: Genomics of Drug Sensitivity in Cancer
NSCLC: Non-Small Cell Lung Cancer

## References

[1] Siegel RL, Giaquinto AN, Jemal A. Cancer statistics, 2024. CA Cancer J Clin. 2024;74(1):12–49. 10.3322/caac.21820 38230766

[2] Shi J, et al. Somatic genomics and clinical features of lung adenocarcinoma: a retrospective study. PLoS Med. 2016;13(12):e1002162. 10.1371/journal.pmed.1002162 PMC5140047 27923066

[3] Xu S, et al. Impact of genetic alterations on outcomes of patients with stage I nonsmall cell lung cancer: An analysis of the cancer genome atlas data. Cancer Med. 2020;9(20):7686–94. 10.1002/cam4.3403 PMC7571826 32857918

[4] Imielinski M, et al. Mapping the hallmarks of lung adenocarcinoma with massively parallel sequencing. Cell. 2012;150(6):1107–20. 10.1016/j.cell.2012.08.029 PMC3557932 22980975

[5] Lim ZF, Ma PC. Emerging insights of tumor heterogeneity and drug resistance mechanisms in lung cancer targeted therapy. J Hematol Oncol. 2019;12(1):134. 10.1186/s13045-019-0818-2 PMC6902404 31815659

[6] Calvayrac O, et al. Molecular biomarkers for lung adenocarcinoma. Eur Respir J. 2017;49(4):1601734. 10.1183/13993003.01734-2016 28381431

[7] Hetz C, Papa FR. The unfolded protein response and cell fate control. Mol Cell. 2018;69(2):169–81. 10.1016/j.molcel.2017.06.017 29107536

[8] Hetz C, Zhang K, Kaufman RJ. Mechanisms, regulation and functions of the unfolded protein response. Nat Rev Mol Cell Biol. 2020;21(8):421–38. 10.1038/s41580-020-0250-z PMC8867924 32457508

[9] Fu XL, Gao DS. Endoplasmic reticulum proteins quality control and the unfolded protein response: the regulative mechanism of organisms against stress injuries. BioFactors. 2014;40(6):569–85. 10.1002/biof.1194 25530003

[10] Urra H, et al. Endoplasmic reticulum stress and the hallmarks of cancer. Trends Cancer. 2016;2(5):252–62. 10.1016/j.trecan.2016.03.007 28741511

[11] Zhang SX, et al. The endoplasmic reticulum: homeostasis and crosstalk in retinal health and disease. Prog Retin Eye Res. 2024;98:101231. 10.1016/j.preteyeres.2023.101231 PMC11056313 38092262

[12] Raymundo DP, et al. Pharmacological targeting of IRE1 in cancer. Trends Cancer. 2020;6(12):1018–30. 10.1016/j.trecan.2020.07.006 32861679

[13] Chen X, Cubillos-Ruiz JR. Endoplasmic reticulum stress signals in the tumour and its microenvironment. Nat Rev Cancer. 2021;21(2):71–88. 10.1038/s41568-020-00312-2 PMC7927882 33214692

[14] Ramirez MU, et al. Endoplasmic reticulum stress pathway, the unfolded protein response, modulates immune function in the tumor microenvironment to impact tumor progression and therapeutic response. Int J Mol Sci. 2019;21(1):169. 10.3390/ijms21010169 PMC6981480 31881743

[15] Schardt JA, et al. Activation of the unfolded protein response is associated with favorable prognosis in acute myeloid leukemia. Clin Cancer Res. 2009;15(11):3834–41. 10.1158/1078-0432.CCR-08-2870 19470730

[16] Wan Q, Jin L, Wang Z. Comprehensive analysis of cancer hallmarks in cutaneous melanoma and identification of a novel unfolded protein response as a prognostic signature. Aging (Albany NY). 2020;12(20):20684–701. 10.18632/aging.103974 PMC7655195 33136551

[17] Su Z, et al. An unfolded protein response-related mRNA signature predicting the survival and therapeutic effect of hepatocellular carcinoma. Comb Chem High Throughput Screen. 2022;25(12):2046–58. 10.2174/1386207325666220204140925 35125080

[18] Fei L, et al. Identification of a novel unfolded protein response related signature for predicting the prognosis of acute myeloid leukemia. Sci Rep. 2025;15(1):6705. 10.1038/s41598-025-91524-9 PMC11861913 40000874

[19] Wang T, et al. Construction and Validation of a UPR-Associated Gene Prognostic Model for Head and Neck Squamous Cell Carcinoma. Biomed Res Int. 2022;2022:8677309. 10.1155/2022/8677309 PMC9192238 35707371

[20] Zhu K, et al. Identification of a novel signature based on unfolded protein response-related gene for predicting prognosis in bladder cancer. Hum Genomics. 2021;15(1):73. 10.1186/s40246-021-00372-x PMC8686253 34930465

[21] Guo H, et al. Immunogenic landscape and risk score prediction based on unfolded protein response (UPR)-related molecular subtypes in hepatocellular carcinoma. Front Immunol. 2023;14:1202324. 10.3389/fimmu.2023.1202324 PMC10348016 37457742

[22] Forsythe N, et al. The unfolded protein response: a novel therapeutic target for poor prognostic BRAF mutant colorectal cancer. Mol Cancer Ther. 2018;17(6):1280–90. 10.1158/1535-7163.MCT-17-0603 PMC5985931 29483217

[23] Leek JT, et al. The sva package for removing batch effects and other unwanted variation in high-throughput experiments. Bioinformatics. 2012;28(6):882–3. 10.1093/bioinformatics/bts034 PMC3307112 22257669

[24] Glasner A, et al. Conserved transcriptional connectivity of regulatory T cells in the tumor microenvironment informs new combination cancer therapy strategies. Nat Immunol. 2023;24(6):1020–35. 10.1038/s41590-023-01504-2 PMC10232368 37127830

[25] Hao Y, et al. Integrated analysis of multimodal single-cell data. Cell. 2021;184(13):3573-3587.e29. 10.1016/j.cell.2021.04.048 PMC8238499 34062119

[26] Li-Fei M, et al. Risk scoring model for lung adenocarcinoma based on PD-L1 related signature reveals prognostic predictability and correlation with tumor immune microenvironment genes was constructed. Front Immunol. 2025;16:1601982. 10.3389/fimmu.2025.1601982 PMC12187644 40568599

[27] Osorio D, Cai JJ. Systematic determination of the mitochondrial proportion in human and mice tissues for single-cell RNA-sequencing data quality control. Bioinformatics. 2021;37(7):963–7. 10.1093/bioinformatics/btaa751 PMC8599307 32840568

[28] Korsunsky I, et al. Fast, sensitive and accurate integration of single-cell data with Harmony. Nat Methods. 2019;16(12):1289–96. 10.1038/s41592-019-0619-0 PMC6884693 31740819

[29] Friedman J, Hastie T, Tibshirani R. Regularization paths for generalized linear models via coordinate descent. J Stat Softw. 2010;33(1):1–22. PMC2929880 20808728

[30] Binder H, Schumacher M. Allowing for mandatory covariates in boosting estimation of sparse high-dimensional survival models. BMC Bioinformatics. 2008;9:14. 10.1186/1471-2105-9-14 PMC2245904 18186927

[31] Ishwaran H, et al. Random survival forests. Ann Appl Stat. 2008;2(3):841–60 20.

[32] Bastien P, Vinzi VE, Tenenhaus M. PLS generalised linear regression. Comput Stat Data Anal. 2005;48(1):17–46.

[33] Bair E, Tibshirani R. Semi-supervised methods to predict patient survival from gene expression data. PLoS Biol. 2004;2(4):E108. 10.1371/journal.pbio.0020108 PMC387275 15094809

[34] Friedman JH. Greedy function approximation: A gradient boosting machine. Ann Stat. 2001;29(5):1189–232 44.

[35] Van Belle V, et al. Support vector methods for survival analysis: a comparison between ranking and regression approaches. Artif Intell Med. 2011;53(2):107–18. 10.1016/j.artmed.2011.06.006 21821401

[36] Liu Z, et al. Machine learning-based integration develops an immune-derived lncRNA signature for improving outcomes in colorectal cancer. Nat Commun. 2022;13(1):816. 10.1038/s41467-022-28421-6 PMC8831564 35145098

[37] Kassambara A, Kosinski M, Biecek P. survminer: Drawing Survival Curves using ‘ggplot2’. R package; 2021. Available from: CRAN.

[38] Blanche P, Dartigues JF, Jacqmin-Gadda H. Estimating and comparing time-dependent areas under receiver operating characteristic curves for censored event times with competing risks. Stat Med. 2013;32(30):5381–97. 10.1002/sim.5958 24027076

[39] Yu G, et al. ClusterProfiler: an R package for comparing biological themes among gene clusters. OMICS. 2012;16(5):284–7. 10.1089/omi.2011.0118 PMC3339379 22455463

[40] Kanehisa M, Goto S. KEGG: Kyoto encyclopedia of genes and genomes. Nucleic Acids Res. 2000;28(1):27–30. 10.1093/nar/28.1.27 PMC102409 10592173

[41] Kanehisa M. Toward understanding the origin and evolution of cellular organisms. Protein Sci. 2019;28(11):1947–51. 10.1002/pro.3715 PMC6798127 31441146

[42] Patil and Indrajeet. Visualizations with statistical details: The 'ggstatsplot' approach. J Open Source Softw. 2021;6(61):3167.

[43] Maeser D, Gruener RF, Huang RS. oncoPredict: an R package for predicting in vivo or cancer patient drug response and biomarkers from cell line screening data. Brief Bioinform. 2021;22(6):bbab260. 10.1093/bib/bbab260 PMC8574972 34260682

[44] Zhao L, et al. A new risk score model based on pyrogenic signatures for the prediction of survival and immune microenvironment features in lung adenocarcinoma. Int J Gen Med. 2023;16:2323–36. 10.2147/IJGM.S411815 PMC10259529 37313044

[45] Chen C, et al. Effects of autophagy-associated genes on the prognosis for lung adenocarcinoma. Transl Cancer Res. 2020;9(3):1947–59. 10.21037/tcr.2020.02.07 PMC8798140 35117541

[46] He J, et al. Fusing WGCNA and machine learning for immune-related gene prognostic index in lung adenocarcinoma: precision prognosis, tumor microenvironment profiling, and biomarker discovery. J Inflamm Res. 2023;16:5309–26. 10.2147/JIR.S436431 PMC10658954 38026246

[47] Gillette MA, et al. Proteogenomic Characterization Reveals Therapeutic Vulnerabilities in Lung Adenocarcinoma. Cell. 2020;182(1):200-225.e35. 10.1016/j.cell.2020.06.013 PMC7373300 32649874

[48] Holder AM, et al. Defining clinically useful biomarkers of immune checkpoint inhibitors in solid tumours. Nat Rev Cancer. 2024;24(7):498–512. 10.1038/s41568-024-00705-7 38867074

[49] Wang S, et al. Machine learning reveals diverse cell death patterns in lung adenocarcinoma prognosis and therapy. NPJ Precis Oncol. 2024;8(1):49. 10.1038/s41698-024-00538-5 PMC10897292 38409471

[50] Inigo JR, Chandra D. The mitochondrial unfolded protein response (UPR(mt)): shielding against toxicity to mitochondria in cancer. J Hematol Oncol. 2022;15(1):98. 10.1186/s13045-022-01317-0 PMC9306209 35864539

[51] Ding D, et al. Machine learning developed an immune evasion signature for predicting prognosis and immunotherapy benefits in lung adenocarcinoma. Front Cell Dev Biol. 2025;13:1622345. 10.3389/fcell.2025.1622345 PMC12222150 40612112

[52] Huang D, et al. The multi-omics analysis identifies a novel endoplasmic reticulum stress and immune related genes signature in lung adenocarcinoma. Discov Oncol. 2025;16(1):1190. 10.1007/s12672-025-03033-w PMC12214066 40591103

[53] Unal B, et al. Targeting IRE1α reprograms the tumor microenvironment and enhances anti-tumor immunity in prostate cancer. Nat Commun. 2024;15(1):8895. 10.1038/s41467-024-53039-1 PMC11480464 39406723

[54] Sinjab A, et al. Resolving the spatial and cellular architecture of lung adenocarcinoma by multiregion single-cell sequencing. Cancer Discov. 2021;11(10):2506–23. 10.1158/2159-8290.CD-20-1285 PMC8487926 33972311

