# Supplementary material for: Machine learning-driven development of a novel unfolded protein response-related gene signature for predicting lung adenocarcinoma patient prognosis
Source: BMC Cancer. 2026 Jul 4;26:1173. doi: 10.1186/s12885-026-16477-2 (PMC13631987; doi:10.1186/s12885-026-16477-2)
Supplement: Supplementary file 1 [file 12885_2026_16477_MOESM4_ESM.pdf]

Raw Western blot images for Fig. 7F

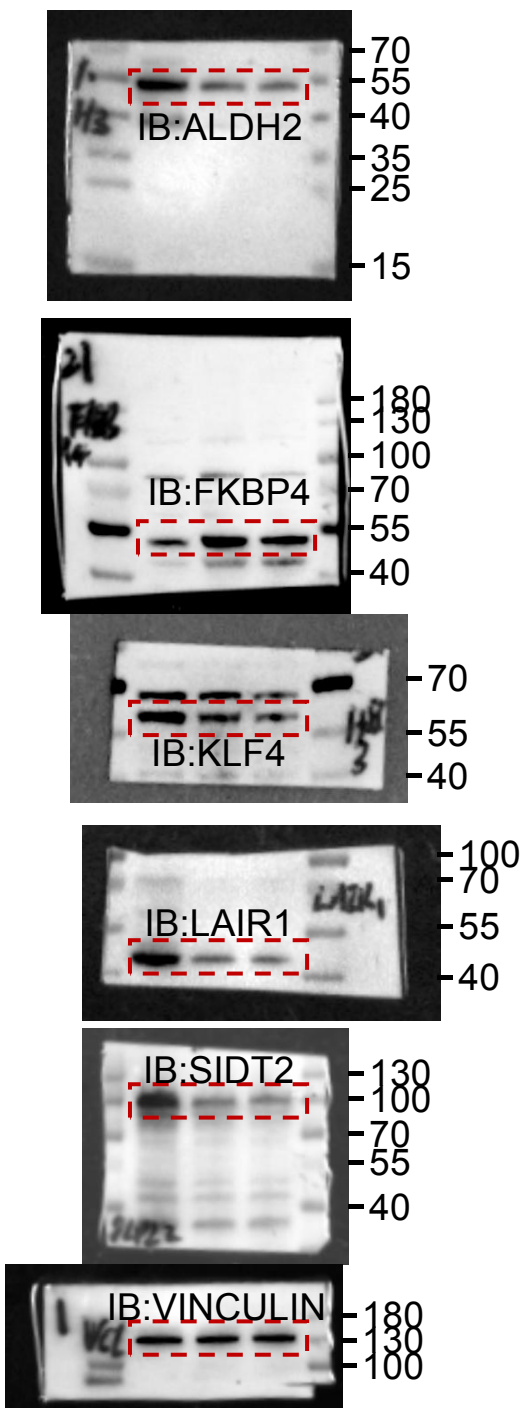

Raw Western blot images for Fig. 7G

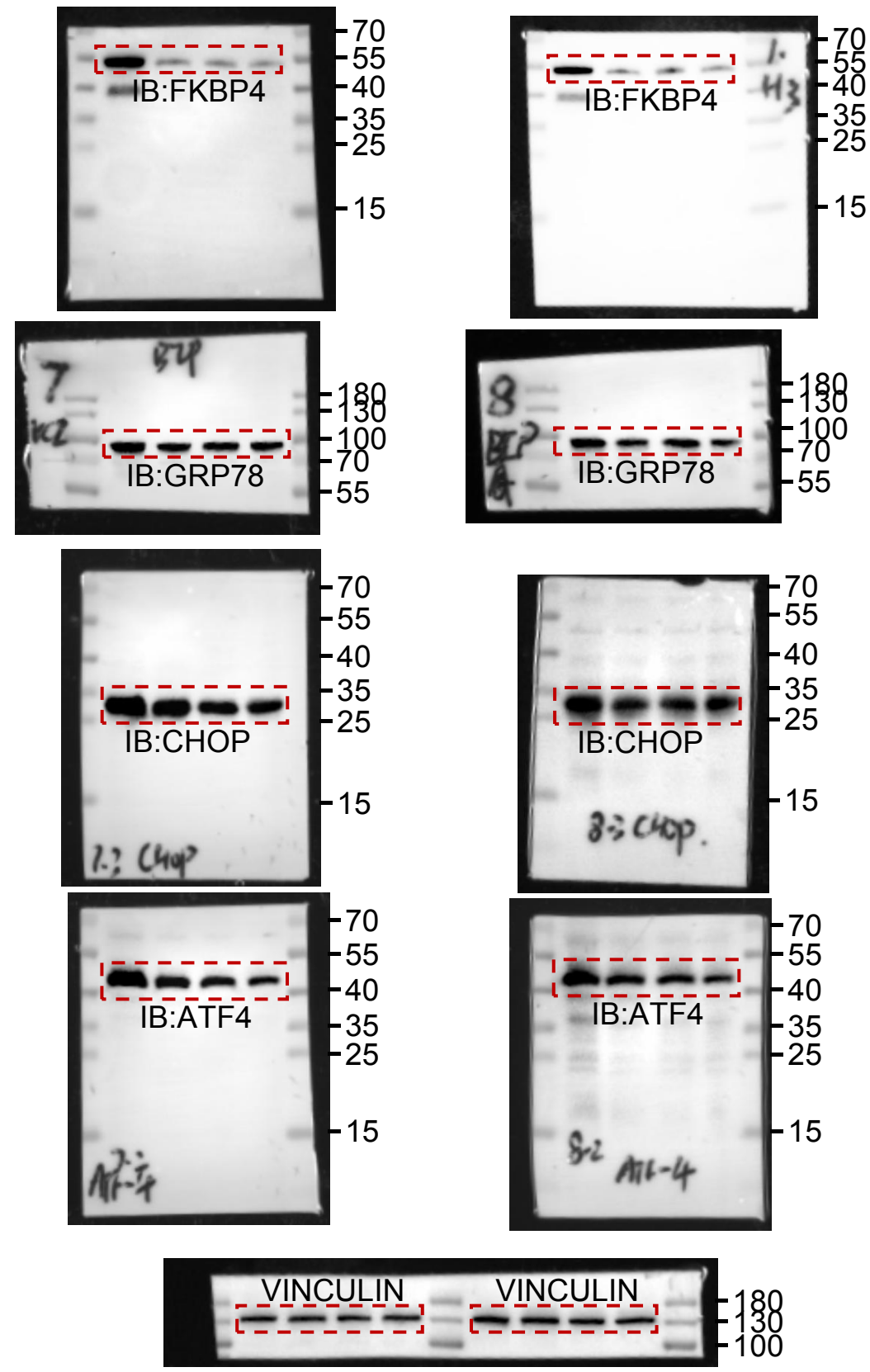

Fig. 7G (A549 cells)

Fig. 7G (H1975 cells)
