# Supplementary material for: Machine learning-driven development of a novel unfolded protein response-related gene signature for predicting lung adenocarcinoma patient prognosis
Source: BMC Cancer. 2026 Jul 4;26:1173. doi: 10.1186/s12885-026-16477-2 (PMC13631987; doi:10.1186/s12885-026-16477-2)
Supplement: Supplementary file 2 [file 12885_2026_16477_MOESM2_ESM.docx]

**Figure S1.** Drug response targeting the unfolded protein response-related signature (UPRRS). (A-H) Drug sensitivity profiles showing the half-maximal inhibitory concentration (IC_50_) values for AZD8186_1918, Camptothecin_1003, Gemcitabine_1190, Irinotecan_1088, Mitoxantrone_1810, MK-2206_1053, Taselisib_1561, and Topotecan_1808, demonstrating differential drug sensitivity between the high- and low-risk UPRRS groups.

**Figure S2.** Sensitivity analysis of model selection using TCGA-only ranking. All 101 candidate models were re-ranked based exclusively on TCGA training and testing cohorts without inclusion of external validation datasets. The selected LASSO + Stepwise Cox model achieved a TCGA-only mean C-index of 0.678. Compared with the top-ranked model under the TCGA-only ranking strategy, the difference in performance was minimal (ΔC-index = 0.006), and the selected model remained consistently among the top-performing pipelines. These results support the stability of model ranking across different evaluation strategies.

**Figure S1.**

**Figure S2.**
