# Supplementary material for: Machine learning-driven development of a novel unfolded protein response-related gene signature for predicting lung adenocarcinoma patient prognosis
Source: BMC Cancer. 2026 Jul 4;26:1173. doi: 10.1186/s12885-026-16477-2 (PMC13631987; doi:10.1186/s12885-026-16477-2)
Supplement: Supplementary file 3 [file 12885_2026_16477_MOESM3_ESM.zip › Jiao_LUAD_Supplement Materials/Jiao_LUAD_EthicsApproval_TDLL20250707.pdf]

INSTITUTIONAL REVIEW BOARD's DECISION

**Assigned No.**	HG-202507-07
**Date**	Jul 9, 2025
**Protocol Title**	A Novel Signature for Predicting Lung Adenocarcinoma Prognosis: Machine - Learning - Driven and Unfolded Protein Response - Associated
**Principal investigators/ Institute**	Rui Jiao/ Department of Thoracic Surgery, the Second Affiliated Hospital of Air Force Medical University<br>Xiaolong Yan/ Department of Thoracic Surgery, the Second Affiliated Hospital of Air Force Medical University

Recommendations of the Institutional Review Board:

All subjects gave their informed consent for inclusion before they participated in the study. The study was conducted in accordance with the Declaration of Helsinki, and the protocol was approved by the Institutional Review Board of Tangdu Hospital, Air Force Medical University.

Decision for this Protocol and has been:

- ☑ Approved
- ☐ Approved with Recommendation
- ☐ Disapproved
- ☐ Termination/Suspense

**Review of Revised Application**	☐ Yes ☑ No
**Continuing Review**	☐ Yes ☑ No<br>If yes, how often to review?<br>☐ Three months ☐ Six months<br>☐ Twelve months

Chairperson: Helong Zheng

Date: Aug 27, 2025

Institutional Review Board, Tang Du Hospital,

Air Force Medical University
