# Supplementary material for: Machine learning-driven development of a novel unfolded protein response-related gene signature for predicting lung adenocarcinoma patient prognosis
Source: BMC Cancer. 2026 Jul 4;26:1173. doi: 10.1186/s12885-026-16477-2 (PMC13631987; doi:10.1186/s12885-026-16477-2)
Supplement: Supplementary file 3 [file 12885_2026_16477_MOESM3_ESM.zip › Jiao_LUAD_Supplement Materials/Jiao_LUAD_Supplement1_PrimerSequences.docx]

ALDH2

FKBP4

KLF4

LAIR1

SIDT2

- **GAPDH-F: TGACTTCAACAGCGACACCCA**
- **GAPDH-R: CACCCTGTTGCTGTAGCCAAA**
